# Development of a Mobile Application Platform for Self-Management of Obesity Using Artificial Intelligence Techniques

**DOI:** 10.1155/2021/6624057

**Published:** 2021-08-27

**Authors:** Sylvester M. Sefa-Yeboah, Kwabena Osei Annor, Valencia J. Koomson, Firibu K. Saalia, Matilda Steiner-Asiedu, Godfrey A. Mills

**Affiliations:** ^1^Department of Computer Engineering, University of Ghana, P.O. Box LG, 77 Legon, Ghana; ^2^Department of Electrical and Computer Engineering, Tufts University, Medford, MA 02155, USA; ^3^Department of Nutrition and Food Science, University of Ghana, P.O. Box LG134, Legon, Ghana

## Abstract

Obesity is a major global health challenge and a risk factor for the leading causes of death, including heart disease, stroke, diabetes, and several types of cancer. Attempts to manage and regulate obesity have led to the implementation of various dietary regulatory initiatives to provide information on the calorie contents of meals. Although knowledge of the calorie content is useful for meal planning, it is not sufficient as other factors, including health status (diabetes, hypertension, etc.) and level of physical activity, are essential in the decision process for obesity management. In this work, we present an artificial intelligence- (AI-) based application that is driven by a genetic algorithm (GA) as a potential tool for tracking a user's energy balance and predicting possible calorie intake required to meet daily calorie needs for obesity management. The algorithm takes the users' input information on desired foods which are selected from a database and extracted records of users on cholesterol level, diabetes status, and level of physical activity, to predict possible meals required to meet the users need. The micro- and macronutrients of food content are used for the computation and prediction of the potential foods required to meet the daily calorie needs. The functionality and performance of the model were tested using a sample of 30 volunteers from the University of Ghana. Results revealed that the model was able to predict both glycemic and non-glycemic foods based on the condition of the user as well as the macro- and micronutrients requirements. Moreover, the system is able to adequately track the progress of the user's weight loss over time, daily nutritional needs, daily calorie intake, and predictions of meals that must be taken to avoid compromising their health. The proposed system can serve as a useful resource for individuals, dieticians, and other health management personnel for managing obesity, patients, and for training students in fields of dietetics and consumer science.

## 1. Introduction

Obesity is defined by the World Health Organization (WHO) as a chronic medical condition whereby excess body fat is accumulated resulting in health impairment [1]. Obesity can be traced to a number of factors such as unhealthy eating habits, genetic factors, physical inactivity, and increasing availability of high fatty foods as well as a low level of awareness of the effects of food intake on human health and physical wellbeing [2, 3]. The prevalence of obesity among children, youth, and adults is increasing at an alarming rate in many parts of the world with its associated health issues such as heart diseases, high blood pressure, cancer, and, in some circumstances, low self-esteem [4–6]. The WHO records show that in 2016, more than 1.9 billion adults were reported to be overweight, and out of this number, 650 million were found obese [1]. Similarly, a 2017 report on obesity in Ghana shows that the rate of obesity among adults has surged from less than 2% to approximately 13.6% [7]. As food becomes affordable and economic conditions improve, coupled with changes in lifestyle, the tendency for these figures to further increase, especially in the developing world will be high [7]. Although conscious efforts are being made by individuals to reduce excess body fat through physical exercises and weight management programs, obesity rates continue to rise. Obesity management strategies face a number of obstacles due to environmental, biological, and behavioral factors. Research studies have shown that sustainable behavioral changes to improve health require real-time, personalized feedback that is tailored to the individual's needs. The emergence of smart mobile devices, application software, and the Internet of Things (IoT) has advanced the development of mobile health (mHealth) solutions for real-time data collection, processing, and user feedback to improve health outcomes.

In this paper, we present a mobile application system for obesity management. The platform operates on smart mobile devices and a web-based platform. The heart of the system is an AI engine that uses parameters such as the user activity level, health status (diabetes and level of cholesterol), age, gender, weight, and height, to advise users on specific meal plans to meet daily caloric and macronutrient requirements. Preliminary performance analysis of the system was conducted on a group of users to validate the operational capabilities and features of the system.

The paper is organized as follows.  Section 2 presents a short overview of research work on mobile and web-based solutions for obesity management.  Section 3 outlines the design and development of the mobile application system, including software design, food database design for food composition and calorie model, and the AI engine. Results are presented in Section 4.  Section 5 presents a discussion and future directions for the research work.

## 2. mHealth Solutions for Obesity Management

A variety of technology-based health management tools and services targeting obesity control have emerged over the past few years [8–13]. Most of these have come in the form of mobile- and web-based solutions. These tools enable the user to provide basic information on foods taken or to be taken and the system estimates the calories remaining to meet the needs for the day.

Alloghani proposed a mobile-based health monitoring application for obesity management that focused primarily on children, using IoT technology [8]. The management system has features that facilitate remote tracking and monitoring of the user by health personnel and parents. The authors evaluated the mobile application using a sample size of 144 and correlation analysis conducted proves high levels of correlation measure of variables such as trust, security, ease of use, and usefulness.

Huang also proposed a dynamic healthcare solution for weight management based on self-regulation [9]. The application enables a user to self-monitor parameters such as meal taken, physical activity, and body weight and transmit the objective data in real-time to a secure web server.

In the work of Jeon and Park, a smart mobile application for clinical-guideline-based obesity management was developed [10]. This was based on knowledge from clinical practice guidelines and consultation with experts. The extracted clinical knowledge was used to design an algorithm for the management of obesity. Test results revealed overall proficiency and efficiency scores of 88.0% and 69.1%, respectively, for the algorithm. In another approach, Barnet proposed an integrated health application system that monitors weight loss and maintenance behavior [11]. The application provides a platform that connects dieticians with patients in a face-to-face consultation and extends the relationship through patients' regular progress updates for sustained behavior change. Much as these remedial measures provide solutions towards the management of obesity in different ways, incidentally, all these systems only monitor the amount of calories that are expended by the user and provide additional information on the remaining amount of calories required to meet the daily energy needs of the user.

So far, the existing obesity management systems do not provide a prediction mechanism for foods that could be taken to meet macronutrient needs. Furthermore, since users are conscious of issues such as cholesterol levels, diabetes, high blood pressure, and hypertension, it will be helpful if these parameters could also be factored in the prediction model of foods for the user to further minimize the problem of health issues of obesity.

## 3. Materials and Methods

### 3.1. System Architecture and Operation

**Figure 1:**
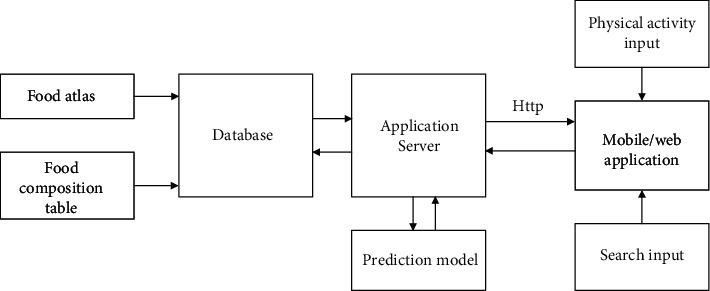
Architectural flow diagram of the obesity management system.

The architectural flow diagram of the proposed obesity management system in Figure 1 consists of four functional systems as database systems for foods and physical activity and levels, calorie computation engine, prediction model, and user application system. These four components are categorized into front-end and back-end applications. The front-end application consists of the user application interface and the calorie computation engine while the back-end contains the database systems and the prediction model. The food database contains locally known basic foods in Ghana, and this was developed using data sources from the Food Composition Table of the Food and Agricultural Organization (FAO) [14] for food decomposition quantification and the Food Atlas [15] for food volume estimation. The calorie computation engine contains a mechanism for decomposition and computation of calorie contents of foods and energy quantification of the physical activity levels (or exercises) of the user. The physical activity database was developed using data from the Hospital for Special Surgery (HSS), USA, [16] on categories of exercises and their levels. The prediction model contains the AI engine for searching for possible foods and meals required to meet the daily calorie and macronutrient needs of a user as well as the desired health conditions of the user. The user application, which operates on both mobile- and web-based platforms, provides the interface for the user to interact with the system by recording food intake and activity tracking. Food intake is recorded by direct selection by the user from a culturally adapted food database. At the front-end, a user provides information via the interface, and other relevant supporting data required for the computation are fetched from the back-end (database) and passed on to the calorie computation engine. The output of the calorie computation engine is then synchronized with the back-end system. Where the need for prediction is required, the prediction model is activated for operation and its output is synchronized with the back-end.

### 3.2. Assumptions for the Obesity Management System

Generally, computing calories, monitoring and managing the calories can be quite complicated since the processes involve a number of parameters and complexities. In order to design and develop a practical and functional obesity management system with the goal to minimize the problem of obesity, we considered the following assumptions:

- Individual users require a fixed amount of calories per day, and this amount must be maintained relatively constant for a healthy condition through replenishments from food
- Amount of calories acquired by users can decline with metabolic activities and other physical activities such as exercises and sleep, which can be monitored, measured, and quantified to estimate the energy content associated with that activity. It is worth stating here that physical activities such as talking, reading, and laughing, which cannot be evaluated, though contributes to energy expenditure were not factored into the development
- At the end of 24 hours each day, any excess amount of calories remaining in the body is nullified and reset to zero. It is recognized, however, that there will be some amount of residual energy at the end of 24 hours each day, but since this residual value cannot be adequately quantified as part of the existing energy, it was assumed to be zero. This certainly can contribute to excess calories and subsequently affect the performance of the system

### 3.3. Data Acquisition and Processing

The first step in the design and development of the obesity management model is the acquisition of food information including the composition of the meal, amount, and volume. For example, a plate of chicken salad at a restaurant may contain a variety of different components and a specific amount and volume or size of salad served. The data required was derived from the FAO Food Composition Table for West Africa [14]. This data set contains a collection of varieties of basic food items such as vegetables, tubers, legumes, fruits, fish, crustaceans, meats, and spices and their corresponding nutritional contents. These food items were structured under the different categories and used in various combinations to constitute complete meals based on preferred recipes. In order to estimate the quantity and volume of foods, the Food Atlas [15] was used. The Food Atlas contains a list of food measures or standard metric sizes for serving food items in grams. The acquired data sets from Food Composition Table and the Food Atlas were pre-processed using a Python Pandas library.

The second stage of the process is the estimation of user energy balance. Energy intake is estimated by retrieving data on the calorie contents of food and beverage items registered in the mobile platform by the user. Energy expenditure is computed by estimating the number of calories expended from physical activity. These estimates are used to compute the user's energy budget and calorie deficits based on the standard daily calorie needs of the user, which is a function of gender and age. To achieve this step, the given user food information is first decomposed into their macronutrients (proteins, carbohydrates, fats, etc.) in grams, and these are used to determine the calorie content of the foods using the Atwater factor [17].  Table 1 shows a sample meal either consumed or intended to be consumed by a user at a time *t*, in the day (example, at 09 : 00 am). The total amount of calories *Z*_*T*_ from this meal is determined as

(1) $$\begin{array}{c} Z_{T}=\overset{M}{\underset{k=1}{\sum }}N_{k}f_{k}\left(4p_{k}+4c_{k}+9h_{k}\right), \end{array}$$

where *k* is the number of individual food items constituting the meal (where *k* = 1, 2, ⋯, 6); *N*_*k*_ is the number of servings of the *k*th food item of the meal; *p*_*k*_, *c*_*k*_, and *h*_*k*_ denote the protein, carbohydrate, and fat content of the meal, respectively; and *f*_*k*_ is the food volume scale factor which is given by expression:

(2) $$\begin{array}{c} f_{k}=\frac{q_{k}}{100}, \end{array}$$

where *q*_*k*_ is the Food Atlas weight measure. Now, to find the average daily calorie needs of a user, we used the Basal Metabolic Rate (BMR) and the user's physical activity level (PAL), which expresses the daily physical activity as a number. These levels are categorized by the World Health Organization (WHO) in terms of level of activeness as extremely inactive, sedentary, moderately active, vigorously active, and extremely active. This threshold calorie requirement is fixed for different age ranges and gender, which is technically expected to be maintained throughout the day. Exceeding this daily prescribed threshold value contributes to the problem of obesity.

The BMR, for a user of any age range and gender, is calculated using the Mifflin-St Jeor equation [18], which is defined as

(3) $$\begin{array}{c} \rho =10W+6.25H-5\alpha +s, \end{array}$$

where *W* denotes the weight of the user in kilograms, *H* is the height in centimeters, *a* is the age in years, and *s* is a measure for the gender (for males, *s* = +5; and for females, *s* = −161). Using this approach for the calorie requirements gives a more accurate estimated value in comparison with values determined from the Estimated Energy Requirements (EER) of the United States Department of Agriculture (USDA) [19].

**Figure 2:**
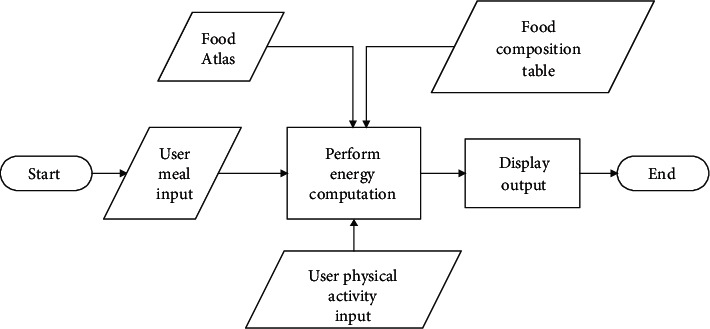
Flow diagram of the calorie extraction and estimation operation.

The amount of energy expended by a user from physical activity (exercise) such as walking and the level of the rigor such as moderate, fast, slow, and extra fast is computed using the expression in equation (4) as [20]

(4) $$\begin{array}{c} E=0.175\beta Wt, \end{array}$$

where *W* denotes the weight of the user, in kilograms, *t* is the duration of the physical activity done in minutes, and *β* is the metabolic equivalent of the exercise, which takes into account, the level of rigor of the activity such as slow, fast, and moderate. Data provided by the HSS was used in the estimation of the expended energy attributable to the exercise. The flow process for the calorie computation is depicted in Figure 2.

### 3.4. Genetic Algorithm Model Formulation

The meal prediction operation was modeled as an optimization problem. The process involves searching from a large space of foods with various combinations, and the solution of the task falls under the category of meals with calories less than or equal to the users' current calorie deficit. Based on the nature of the optimization problem and exhaustive search operation, the Genetic Algorithm (GA) technique [21] was considered a suitable model for adoption.

GAs are a type of optimization algorithm primarily employed to find optimal solutions to computational problems that maximize or minimize a particular function. GAs search for models based on the natural and genetic selection process, to optimize a population and give a solution that is optimal in the sense of a fitting function. In the GA process, no extra information is required about the given problem, a feature that allows GAs to find solutions to problems that are not possible with other optimization approaches, especially with issues of linearity, continuity, and incomplete or imperfect information or limited computational capacity. GAs are less susceptible to getting trapped in the local optima of the optimization function since they make the search process a multiple directional operations [22].

**Figure 3:**
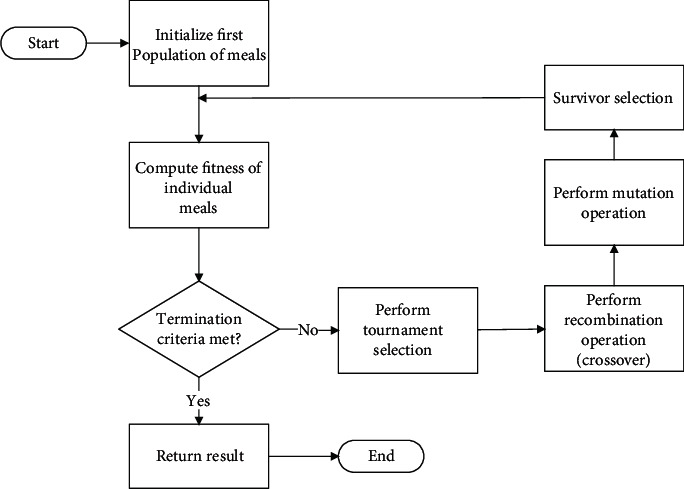
General flow diagram for genetic algorithm model.

The basic components required for the implementation of a GA model for any given problem are the following: (1) a fitness function of the problem to be solved by the optimization algorithm, (2) a population of chromosomes of the candidate solutions to the problem for the algorithm to solve, (3) selection of the chromosomes that will reproduce, (4) a crossover to produce the next generation of chromosomes, and (5) random mutation of chromosomes in the new generation. The general process for GA feature selection for model development is illustrated in the flow diagram in Figure 3. The GA begins with a randomly chosen assortment of chromosomes, which serves as the first generation or initial population, and each chromosome in the population is then evaluated by the fitness function to test how well it solves the problem. The selection operator then chooses some of the chromosomes for reproduction based on a defined probability distribution. The selection, crossover, and mutation process continue until the number of offspring becomes equal to the initial population. Thus, the second generation is composed entirely of new offspring, and the first generation is completely replaced.

The performance of a GA is primarily dependent on the method used for encoding the candidate solutions into chromosomes and the parameter that the fitness function is measuring. Important control parameters that define the performance of the GA include the size of the population, probability of crossover, probability of mutation, and number of iterations. Parameterization of a GA model design, therefore, depends on the problem definition, which relates to the values that are attributed to the parameters, and the choice of implementation of the operators. To design the GA model for the meal prediction system, two key components, which are the model control parameter and the fitness model, were first established.

#### 3.4.1. Model Control Parameter Selection

The genotype representation of a chromosome employed was codified using value encoding of all food components in the meal. This encoding was chosen because the algorithm optimizes against real values and using binary encoding will introduce an overhead during encoding and decoding phases. The total calories and macronutrients in a meal change with respect to variations in its serving size as well as the number of servings (defined in equation (1)). The “fit individuals” required for the algorithm were determined based on tournament selection. For a specified tournament size, the fit individuals are selected from a mating pool which is based on the total calories for a meal. Two parents from the current generation are then selected from a sorted list of all the fit individuals in the population. Based on the selection of individuals, a recombination operation is performed on the “fit parents” to meet a criterion for the crossover parameter. This operation produced offspring (one or more) that share the same characteristics of the parents. The corresponding genes from the parents are then crossed over to the child. The mutation process and operation ensure that the population is supplied with new material throughout the search process [22]. For the meal prediction model, the number of food servings and the size of food servings of the child meals are altered in this step. To determine the optimal values for the population size (or number of chromosomes) and the mutation rate required to achieve good performance, a number of sensitivity analyses were carried out.

#### 3.4.2. Model Fitness Function Evaluation

Using information from equations (1) and (2), we define the fitness function of the individual chromosomes based on the following objective functions:

(5) $$\begin{array}{c} y_{1}=Q+E^{*}-Z_{1}-d_{1}\leq 0, \end{array}$$

(6) $$\begin{array}{c} y_{k}=d_{k-1}+E^{*}-Z_{k}-d_{k}\leq 0. \end{array}$$

The parameter *Q* denotes daily energy or calorie requirement for an individual, *Z* is the amount of calories derived from a given meal, *d* represents the calorie deficit, and *E*^∗^ is the energy expended due to activity. Equation (5) represents the initial objective function, while equation (6) defines the subsequent runs of objective functions, for *k* = 2, 3, 4⋯. The constraints on the objective functions are

(7) $$\begin{array}{c} \overset{M}{\underset{k=1}{\sum }}p_{k}-P\leq 0, \end{array}$$

(8) $$\begin{array}{c} \overset{M}{\underset{k=1}{\sum }}h_{k}-F\leq 0, \end{array}$$

(9) $$\begin{array}{c} \overset{M}{\underset{k=1}{\sum }}c_{k}-C\leq 0, \end{array}$$

(10) $$\begin{array}{c} 0\leq g_{k}\leq 55, \end{array}$$

where *p*_*k*_, *h*_*k*_, and *c*_*k*_ are the individual macronutrients of protein, fats, and carbohydrate contained in a given meal or food, and *P*, *F*, and *C*, respectively, denote the current amounts of protein, fats, and carbohydrate deficit, and *g*_*k*_ represents the glycemic index contained in a meal. For the case of a user with a condition of diabetes, equation (10) is implemented as a constraint.

To use the GA model for meal prediction, we first formulated a solution that has total calorie or macronutrient values defined in a range bound by the calorie deficit or each macronutrient deficit. For example, if a user has a daily calorie target requirement of 2400 kcal and has already consumed 400 kcal, then a valid solution will have total energy with an upper bound value of 2000 kcal. Similarly, each macronutrient contained in a given meal is bounded using a similar analysis. Following the establishment of a possible optimization solution, the chromosomes are then encoded using the real values, which we encapsulated in a data structure format for convenience. The attributes of this data structure are represented as:

(11) $$\begin{array}{c} \begin{array}{c} X_{k}=\left\{c_{k},p_{k},h_{k},g_{k},N_{k},f_{k},\left[x_{k1,}x_{k2\cdots }\right]\right\},\\ x_{ki}=\left\{c_{ki},p_{ki},h_{ki},g_{ki},N_{ki},f_{ki}\right\}, \end{array} \end{array}$$

where *X*_*k*_ denotes a chromosome, where each chromosome has a list of meal components, and *x*_*ki*_ represents an additional meal component.  Table 2 shows sample chromosome representation for given set of meal.

To configure the GA model requires establishing the number of chromosomes in a population. In our model, the initial chromosome population is established by randomly selecting required meals from the food database, where each meal is created using a combination of the Food Atlas and the Food Composition Table. The objective function value for the *j*th chromosome, *f*(*j*), that was produced at initialization was computed. This was computed for each of the initial chromosomes. We then proceeded to obtain the *fittest* chromosomes of the initial population using tournament selection, where a *fit* chromosome satisfies equations (5) and (6). The *fittest* chromosomes have a higher probability of selection for the next generation. To determine the fitness probability, we first computed the fitness for each chromosome and formulated the probability for the chromosome to be chosen for reproduction as *P*[*j*] = *Fitness*[*j*]/total sum of the fitness for the number of chromosome population, where *Fitness*[*j*] = 1/(1 + *f*[j]). We thus computed the energy *Z*_*k*_ of chromosome *X*_*k*_ using equation (1). Parent chromosome which will mate is randomly selected and the number of mate chromosomes is controlled using crossover rate or probability. If the crossover criteria for the chromosome generation is met, then a single-point crossover is performed; otherwise, no crossover is performed for that generation. The crossover operation swaps the gene that is responsible for additional meal components (*x*_*k*1_ ⋯ *n*) for the selected parents. For example, suppose we have two parents *X*_1_ and *X*_2_, denoting two sets of complete meals of a user, which are defined as

(12) $$\begin{array}{c} \begin{array}{c} X_{1}=\left\{c_{1},p_{1},h_{1},g_{1},N_{1},f_{1},\left[x_{11,}x_{12,}x_{13}\right]\right\},\\ X_{2}=\left\{c_{2},p_{2},h_{2},g_{2},N_{2},f_{2},\left[x_{21,}x_{22}\right]\right\}. \end{array} \end{array}$$

Then, the crossover operation will result in two offspring chromosomes as

(13) $$\begin{array}{c} \begin{array}{c} X_{12}=\left\{c_{1},p_{1},h_{1},g_{1},N_{1},f_{1},\left[x_{21,}x_{22}\right]\;\right\},\\ X_{21}=\left\{c_{2},p_{2},h_{2},g_{2},N_{2},f_{2},\left[x_{11,}x_{12,}x_{13}\right]\right\}. \end{array} \end{array}$$

Using the chromosome representation of meals in Tables 2 and 3 as parents *X*_1_ and *X*_2_, respectively, in the current generation; then, the two offsprings in the next generation will be obtained using equation (13). Thus, the resulting child chromosomes that will be obtained from the parents *X*_1_ and *X*_2_ are shown in Tables 4 and 5.

The number of chromosomes that have mutations in a population is determined by the mutation rate parameter. The mutation operation is done by replacing the gene at random position with new value. For the mutation process, we first calculated the total length of genes in the population, by finding the product of the number of genes in the chromosome and the number of population. Random integers were then generated between 1 and the maximum gene length. If the generated random number is found to be smaller than the mutation rate variable, then the position of the gene in the chromosomes is marked. For a given mutation rate variable, we changed the serving sizes (*f*_*k*_) and the number of servings (*N*_*k*_) of the children chromosomes as well as their respective additional meal components to determine the optimal solution. Thus, after the mutation process, we then have one iteration or one generation of the GA operation and evaluate the objective function after the first generation to obtain the new chromosomes for the next generation. The new chromosomes followed the same process of evaluation, selection, crossover, and mutation, repeatedly until a predetermined number of generations or stop criterion for the optimization operation. In this work, after running a maximum number of 500 iterations, the GA model produced optimal chromosome solutions.

To establish the best model suitable for adoption for the prediction model, a variety of system control parameters were tested, model control parameters were adjusted, and performance accuracies were measured. The system parameters used for the GA model configuration are

- Size of population: 20, 30, 40, 50, 70, 100
- Number of generations: 500, 2500
- Mutation probability: 0.001, 0.01, 0.08, 0.1, 0.5, 0.9
- Crossover probability: 0.65, 0.9
- Crossover mechanism: single-point and uniform

Following a number of trial runs and performance accuracy measurements (sample results shown in Table 6), a GA model with a population size of 30, mutation probability of 0.01, 500 number of generations, and crossover probability of 0.65 was found suitable with best convergence time and least deviations and was subsequently adopted for the prediction model.

### 3.5. Food Prediction Model

**Figure 4:**
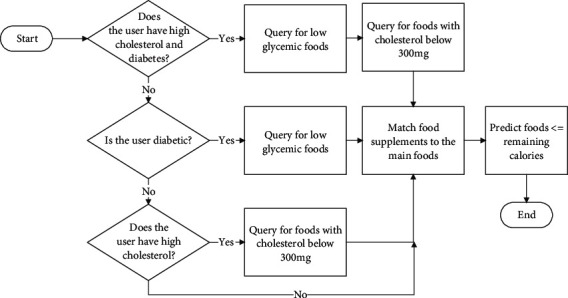
Operational logic diagram for the meal prediction model.

The goal of the food prediction model is to analyze information and make decisions on possible foods and meals required to meet the overall daily calorie and macronutrient needs at any given time and to ensure that these will not exceed the daily deficit. At the same time, the model solution must ensure that the health of the user is not compromised as a result of a bad choice of foods or eating habits. The flow diagram for the prediction process is shown in Figure 4. Information from the health status records of a user such as diabetes and cholesterol conditions are extracted from the registration data for analysis in the model to aid the prediction process. For users with diabetes and high cholesterol conditions, the algorithm predicts foods that contain a low glycemic index and cholesterol content less than 300 mg in accordance with the WHO standard requirements. The prediction model was implemented using the Python Django framework.

### 3.6. User Application Software Design

**Figure 5:**
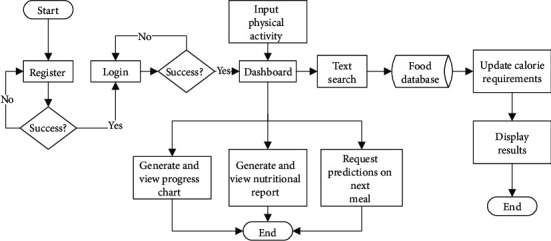
Operational flow diagram for the obesity management system.

The user application was designed as an interactive process to capture information from users as well as provide output to the users. The implemented operational logic flow for the user application is shown in Figure 5. The application has interfaces for user registration, health status information, authentication, and input information on food to be consumed. The current system supports input food selection using a search interface for meals. Options for input foods from sources such as barcode scanning, image processing of scanned text receipts, and image processing of photographed foods have been created and are still undergoing refinements for accuracy.

**Figure 6:**
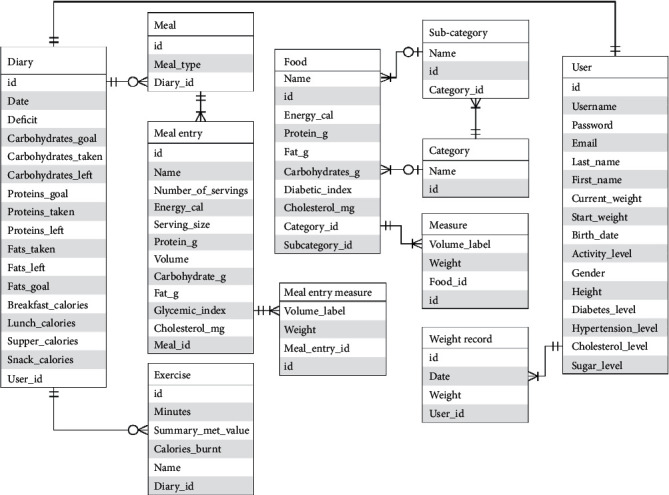
Entity relational diagram for the data relations and management.

**Figure 7:**
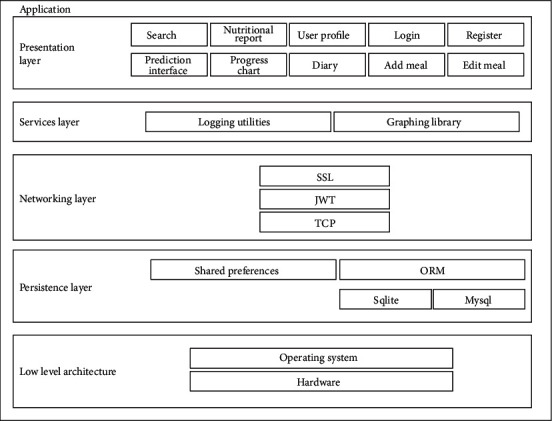
Architectural diagram for obesity management software application.

The user application software was designed for two platforms: mobile-based using Android platform and web-based platform. The mobile-based application was developed using the Model View Presenter (MVP) pattern [23] to allow for code maintainability and efficient code testing. The web-based application, on the other hand, was developed using the Angular2 front-end framework and Model View Controller (MVC) software development pattern [24]. The web-server application hosts both the database system and the prediction model.  Figure 6 shows the entity relational diagram for the management of data in the obesity management system. The database was developed using basic locally known foods in Ghana and was implemented using the MySQL relational database system.  Figure 7 shows the architectural diagram of the software implementation where the presentation layer serves as an interface for the user to access features of the application.

## 4. Results and Discussion

### 4.1. Experimental Setup and Testing

The model was first tested in a numerical simulation environment using the Python programming language. The simulation was followed by an experimental setup to validate the functionality and operational capability of the genetic algorithm to accurately predict information. In the numerical simulation environment, the system was tested using randomly generated data to mimic the information from the input food and other required parameters pertaining to daily calorie and macro-nutrient deficits, and health status.

During the experimental setup, the obesity management system was deployed on the University of Ghana campus network server and tested for functionality, efficiency, operational accuracy limits, and performance. The specifications for the server are Linux Ubuntu 16.0 operating system, RAM of 4 GB, storage capacity of 10 GB, and CPU speed of 2.2 GHz. The application was installed on Samsung Galaxy S6 edge+, which was used for the test. The average connection time required by a user device to access the obesity management system is approximately 201.24 milliseconds and is network dependent. To avoid user congestion problems, load balancing using Nginx was implemented. The experiment was conducted over a period of 40 days using a variety of food selections for breakfast, lunch, snacks, and dinner and individual fitness and health status information. The application was also tested by 30 other individuals comprising different user groups (different age groups, gender, students, individuals, and dieticians).  Table 7 shows a selected list of sample information from user testing.

**Figure 8:**
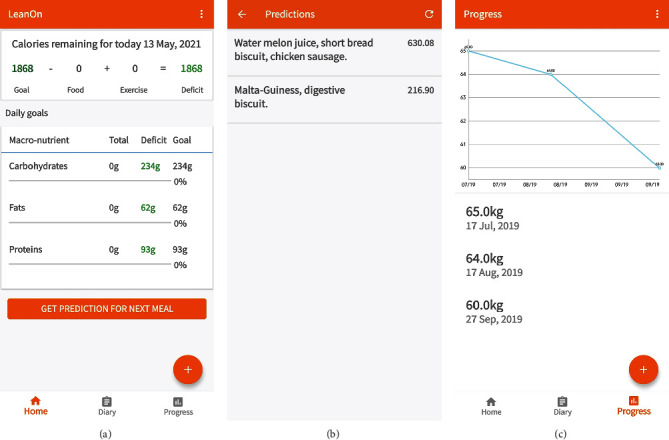
User interface report showing (a) daily calorie and macronutrient summary, (b) meal prediction results, and (c) progress chart of weight loss over time.

**Figure 9:**
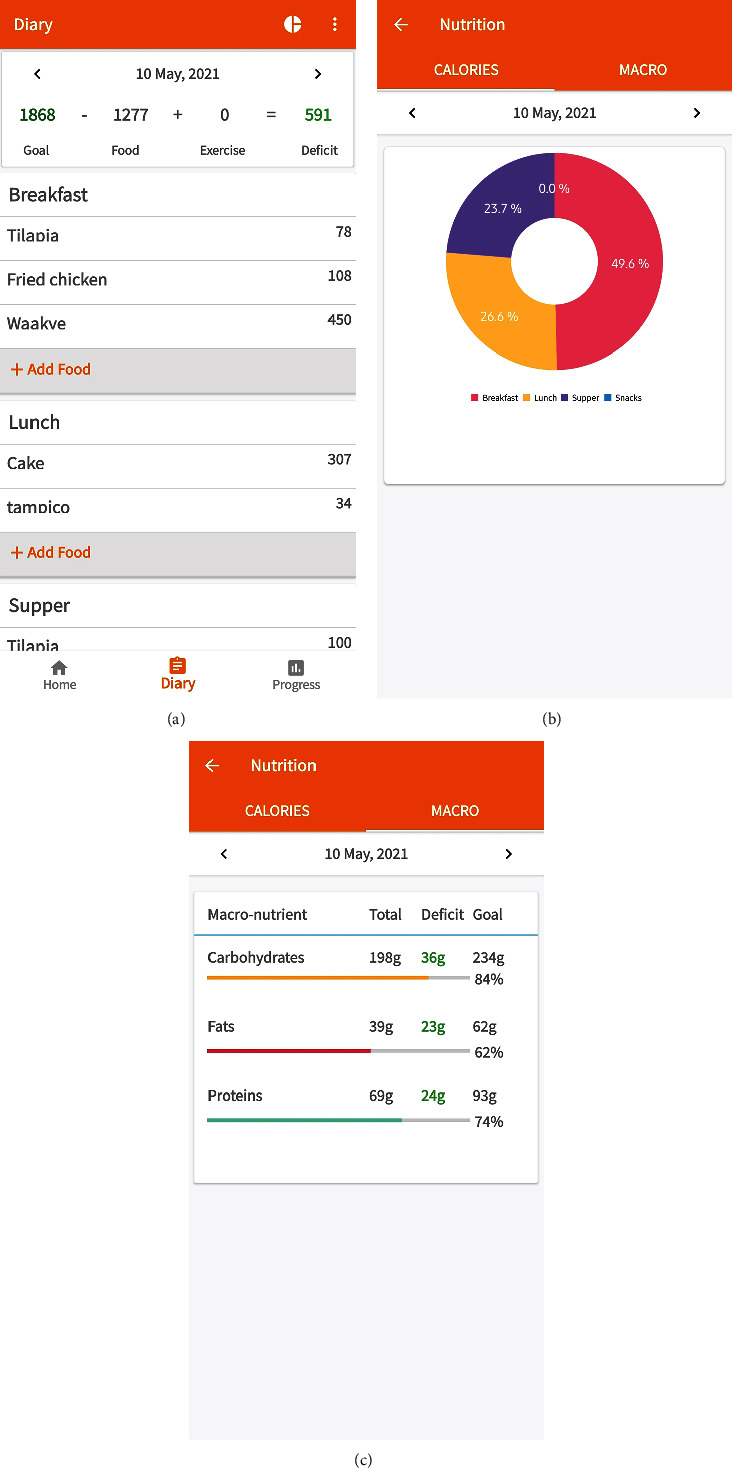
User interface report showing (a) user diary of meal activities, (b) calorie consumption, and (c) macronutrient consumption.

To determine the calorie and macro-/micronutrient needs or deficits over a 24-hour period, a user inputs foods consumed for breakfast, lunch, dinner, and snacks from the food database. This is important for the user to maintain a balance in the calorie requirements for the body. For an existing user of the system, tracking information on weight loss or gain over the period is captured and displayed as well.  Figure 8(a) shows a sample user interface with the desired food input and macronutrient goals. The food selection is done by navigating through a diary (Figure 9(a)) which allows a user to select any variety and combination of foods by searching through the food database. The user then determines the volume or quantity desired for the selected foods, and the total energy is computed and displayed on the user interface. To include physical activity in the computation, a user selects and logs the type of exercise from the physical activity database. Although this provides an estimate, it is worth pointing out that practical data from real-time monitoring of activities such as the physical motion of the user will give more accurate results.

To decide on the next meal intake required to meet all deficits and constraints in calories and macronutrients for any given day and time, the user invokes the prediction algorithm from the user interface.  Figure 8(b) shows a sample user interface for the prediction of possible foods required to meet the deficits and health conditions. From the predicted foods, it can be observed that the recommended foods have total calorie requirements not exceeding the computed deficit and the remaining macronutrients are also not exceeded. The progress chart shows the weight loss or gain with time (Figure 8(c)). The user interface also allows the user to update weight information. Figures 9(b) and 9(c) shows generated sample nutritional report on calories consumed per meal for any given day along with the macronutrients. The daily report serves as a personal health assistant to guide the user on input food choices based on their health status.

### 4.2. Performance Evaluation of the System

The performance of the obesity management system is computed in terms of computational time and prediction accuracy. To determine the system performance, various simulations were run. These simulations involved averaging the convergence time of the genetic algorithm over a hundred runs (shown in Table 3) for various calorie values (1000 kcal, 1600 kcal, 2000 kcal, 2400 kcal, 2800 kcal, and 3200 kcal). These calorie limits were chosen based on the average requirements set by the WHO.

**Figure 10:**
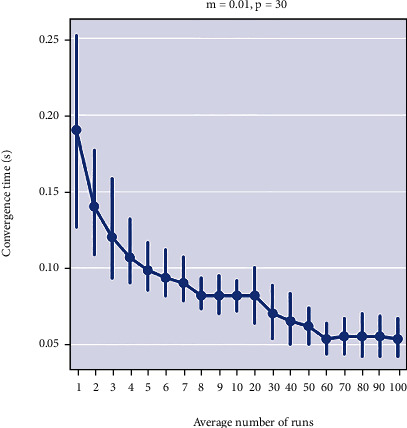
Categorical plot of convergence of algorithm for a mutation rate (*m*) of 0.01 for populations (*p*) of 30.

From the performance results in Table 6, a population size of 30 with a mutation rate of 0.01 gives the best convergence time with the least deviations in predicting for all calorie ranges. In view of that, we adopted a population size of 30, mutation rate of 0.01, number of generations of 500, and uniform single-point crossover with a probability of 0.65.  Figure 10 shows the simulation results for the selected parameters.

A sample run of the configuration in a real-time scenario is shown below.  Table 8 shows the information of the daily needs for a male user within the age range of 23 to 30 years.

**Figure 11:**
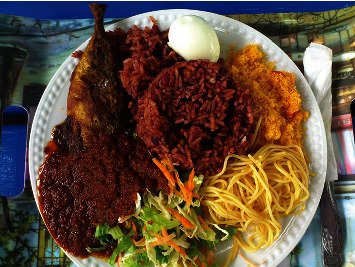
A sample meal selected by a user.

The user has already consumed the meal shown in Figure 11 and Table 9, for breakfast. The user then requests a suggestion for the next meal, shown in Table 10.

## 5. Conclusions

We presented a software-based obesity management solution that can be deployed on both mobile and web-based platforms. The system uses a genetic algorithm as an artificial intelligence engine to predict foods required to meet the calorie and macro-/micronutrient needs of the user. Test results obtained from 30 volunteers who used the system showed that the proposed obesity management platform is able to adequately determine daily energy intake and expenditure and make recommendations to users for future meals. Meal recommendations are designed to ensure users maintain specific nutritional targets to minimize the problems of diabetes and high cholesterol. In addition to self-management strategies, this system can serve as a useful resource for dieticians and other health management personnel for managing patients with the condition of obesity. It can also serve as a training tool for students in the field of dietetics as well as nutrition and consumer science.

A limitation of the current system, however, is the use of HSS predefined levels of physical activity. This data does not give an accurate estimation of energy expenditure by users and, consequently, affects the overall output performance of the system. Two options are currently under consideration for accurate measurement of energy expenditure: (a) integration of data from a mobile app and (b) wireless integration of a wearable device to track physical activity. Moreover, the current system only supports direct user selection of foods to record energy intake. Barcode scanning of packaged foods, data extraction from food receipts, and image processing of photographs of foods are methods being explored in future implementations to record energy intake.

Although the prediction model is able to accurately predict meals to be taken without exceeding the daily calorie and macronutrient needs, relying on the eating patterns of users for prediction to focus meal suggestions towards user preferences may be useful. Future work will focus on a learning agent for this feature.

## Figures and Tables

**Table 1:** Sample meal with various food components and measures.

Food selected	Serving size (*f*)	Number of servings (*N*)
Rice	Soup ladle	3
Fried chicken	Portion	1
Tomato stew	Soup ladle	1
Egg	1 egg	2
Salad	Stew ladle	3
Shito	Teaspoon	1

**Table 2:** Chromosome representation of a meal.

Chromosome	Phenotype	*f*	*N*	*c*	*p*	*h*	*g*	*Z*
*X_1_*	Kenkey(Ga)	285.94	1	25.00	3.50	0.60	35.00	341.41
*x_11_*	Okro soup	100.00	1	4.20	1.90	2.80	30.00	49.60
*x_12_*	Hot pepper	100.00	1	10.70	1.10	48.10	0.00	48.10
*x_13_*	Tilapia	100.00	1	0.00	18.80	2.70	0.00	99.50
			Total					538.61

**Table 3:** Sample chromosome representation of parent meal *X*_2_.

Chromosome	Phenotype	*f*	*N*	*c*	*p*	*h*	*g*	*Z*
*X*_2_	Rice ball	222.39	1	79.60	7.20	0.40	70.00	2340.43
*x*_21_	Palm nut soup	100.00	2	16.20	1.80	46.80	20.00	986.40
*x*_22_	Rabbit	100.00	1	0.00	21.60	21.10	0.00	129.60
*x*_23_	Smoked tuna	100.00	2	0.00	31.80	31.70	90.00	259.80
			Total					3716.23

**Table 4:** Sample chromosome representation of child meal *X*_12_.

Chromosome	Phenotype	*f*	*N*	*c*	*p*	*h*	*g*	*Z*
*X*_12_	Kenkey (Ga)	285.94	1	25.00	3.50	0.60	35.0	341.41
*x*_11_	Palm nut soup	100.00	2	16.20	1.80	46.80	20.0	986.40
*x*_12_	Rabbit	100.00	1	0.00	21.60	21.10	0.00	129.60
*x*_13_	Smoked tuna	100.00	2	0.00	31.80	31.70	90.00	259.80
			Total					1717.21

**Table 5:** Sample chromosome representation of child meal *X*_21_.

Chromosome	Phenotype	*f*	*N*	*c*	*p*	*h*	*g*	*Z*
*X*_21_	Rice ball	222.39	1	79.60	7.20	0.40	70.00	2340.43
*x*_11_	Okro soup	100.00	1	4.20	1.90	2.80	30.00	49.60
*x*_12_	Hot pepper	100.00	1	10.70	1.10	48.10	0.00	48.10
*x*_13_	Tilapia	100.00	1	0.00	18.80	2.70	0.00	99.50
			Total					2537.63

**Table 6:** Average convergence time and deviation for simulations in seconds.

Pop.	*m* = 0.001		*m* = 0.01		*m* = 0.08		*m* = 0.1		*m* = 0.5		*m* = 0.9	
*Size*	*μ*	*σ*	*μ*	*σ*	*μ*	*σ*	*μ*	*σ*	*μ*	*σ*	*μ*	*σ*
*p* = 20	0.0667	0.0372	0.0416	0.0240	0.1467	0.1809	0.1933	0.3137	0.0450	0.0104	0.0383	0.0098
*p* = 30	0.0683	0.0462	0.0533	0.0175	0.0716	0.0337	0.0550	0.0104	0.1633	0.1802	0.1400	0.1447
*p* = 40	0.1183	0.0231	0.8283	1.7459	0.1766	0.1339	0.1000	0.0374	0.0616	0.0116	0.1000	0.0632
*p* = 50	0.1150	0.0484	0.1183	0.0331	0.1566	0.0608	0.4083	0.6014	0.2833	0.3626	0.1066	0.0301
*p* = 70	0.1333	0.0307	1.0550	0.9813	0.6716	0.7598	0.1533	0.0575	0.1766	0.0436	0.1533	0.0472
*p* = 100	0.2366	0.0871	0.2250	0.1320	0.4983	0.6577	0.2016	0.0699	0.2033	0.0578	0.3316	0.1422

**Table 7:** Sample user data collected during the testing process.

Age	Gender	Height (cm)	Start weight (kg)	Current weight (kg)	Diabetes level	Cholesterol level	Hypertension level	Sugar level
23	Male	165.00	65	60	Low	None	None	Low
24	Male	165.00	65	65	None	None	None	Low
20	Male	205.74	65	65	None	High	Low	High
25	Male	165.00	65	63	None	None	None	None
24	Female	167.00	70	65	None	None	None	None
21	Male	172.20	85	85	Medium	Medium	Medium	Medium
35	Female	163.00	55	85	Low	None	Low	None
24	Male	177.00	85	63	Low	Medium	Low	Low
27	Male	181.00	63	85	Low	None	None	Medium

**Table 8:** Sample user data with daily calorie and macronutrient requirements.

Activity level	BMI	BMR	Calorie requirement/kcal	Carbohydrate requirement/g	Protein requirement/g	Fat requirement/g
Little to no exercise	27.13	1841.25	2210	276.25	110.5	73.67

**Table 9:** Sample user data with daily calorie and macronutrient requirements.

Meal entries	Serving size	Number of servings	Calorie content/kcal	Carbohydrate content/g	Protein content/g	Fat content/g
Waakye	Soup ladle	2	450.00	108.00	16.00	12.00
Salad	Stew ladle	2	15.00	3.00	1.00	0.00
Spaghetti	Stew ladle	2	134.00	28.00	6.00	1.00
Gari	Table spoon	2	55.04	13.00	0.00	0.00
Fried fish	1 whole	1	78.00	0.00	15.00	2.00
Tomato Stew	Stew ladle	1	114.60	0.00	13.00	2.00
Egg	1 egg	1	87.00	0.00	6.00	8.00
Shito	Teaspoon	2	83.50	1.00	3.00	8.00
		Total	1017.50	153.00	60.00	33.00

**Table 10:** Sample predicted meals.

Meal entries	Serving size	Number of servings	Calorie content/kcal	Carbohydrate content/g	Protein content/g	Fat content/g
Banku	Small ball	1	334.96	23.80	2.40	0.40
Okro stew	100 g	1	135.60	5.00	2.80	11.60
Tilapia	100 g	1	99.50	0.00	18.80	2.70
Lamb/mutton	100 g	1	256.80	0.00	16.50	21.20
		Total	826.86	28.80	39.70	35.90
Apple juice	240 ml	2	278.40	29.00	0.00	0.00
Wheat bread	1 slice	1	50.51	12.90	2.70	1.20
		Total	328.91	41.90	2.70	33.00

## Data Availability

Access to data may be granted on request.
